# (5*R*)-3-(2-Chloro­acet­yl)-4-methyl-5-phenyl-1,3,4-oxadiazinan-2-one

**DOI:** 10.1107/S1600536811020356

**Published:** 2011-06-04

**Authors:** Ignez Caracelli, Daniel C. S. Coelho, Paulo R. Olivato, Alessandro Rodrigues, Edward R. T. Tiekink

**Affiliations:** a BioMat-Departamento de Física, Universidade Federal de São Carlos, CP 676, 13565-905 São Carlos, SP, Brazil; b Laboratório de Cristalografia, Estereodinâmica e Modelagem Molecular, Universidade Federal de São Carlos, Departamento de Quïmica, CP 676, 13565-905 São Carlos, SP, Brazil; c Universidade de São Paulo, Conformational Analysis and Electronic Interactions Laboratory, Instituto de Química, São Paulo, SP, Brazil; d Departamento de Ciências Exatas e da Terra, Universidade Federal de São Paulo, UNIFESP, Diadema, Brazil; e Department of Chemistry, University of Malaya, 50603 Kuala Lumpur, Malaysia

## Abstract

The 1,3,4-oxadiazinan-2-one ring in the title compound, C_12_H_13_ClN_2_O_3_, is in a distorted half-chair conformation. The phenyl and chloro­acetyl groups occupy axial and equatorial positions, respectively, and lie to the opposite side of the mol­ecule to the N-bound methyl substituent. Mol­ecules are consolidated in the crystal structure by C—H⋯O inter­actions.

## Related literature

For background to 1,3,4-oxadiazin-2-ones, see: Trepanier *et al.* (1968 ▶); Roussi *et al.* (1998 ▶, 1999 ▶, 2000 ▶); Casper *et al.* (2002*a* ▶,*b* ▶); Bonin *et al.* (2006 ▶). For a related structure, see: Zukerman-Schpector *et al.* (2009 ▶). For the synthesis, see: Rodrigues *et al.* (2005 ▶). For conformational analysis, see: Cremer & Pople (1975 ▶).
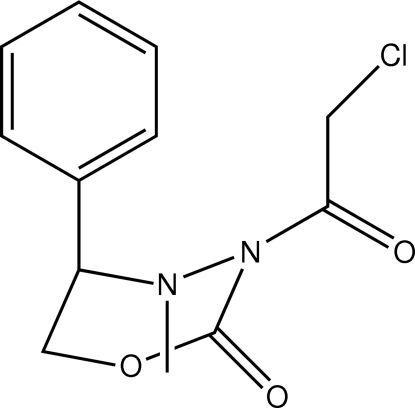


## Experimental

### 

#### Crystal data

C_12_H_13_ClN_2_O_3_
*M* *_r_* = 268.69
Orthorhombic, 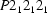

*a* = 9.4862 (2) Å
*b* = 9.6237 (2) Å
*c* = 13.2433 (3) Å
*V* = 1209.01 (5) Å^3^
*Z* = 4
Mo *K*α radiation
μ = 0.32 mm^−1^
*T* = 100 K
0.35 × 0.30 × 0.25 mm

#### Data collection

Bruker APEXII CCD diffractometer
29813 measured reflections
2378 independent reflections
2334 reflections with *I* > 2σ(*I*)
*R*_int_ = 0.026

#### Refinement

*R*[*F* ^2^ > 2σ(*F* ^2^)] = 0.020
*wR*(*F* ^2^) = 0.054
*S* = 1.07
2378 reflections
164 parameters
H-atom parameters constrained
Δρ_max_ = 0.19 e Å^−3^
Δρ_min_ = −0.20 e Å^−3^
Absolute structure: Flack (1983 ▶), 993 Friedel pairs
Flack parameter: 0.01 (5)

### 

Data collection: *APEX2* (Bruker, 2007 ▶); cell refinement: *SAINT* (Bruker, 2007 ▶); data reduction: *SAINT*; program(s) used to solve structure: *SIR97* (Altomare *et al.*, 1999 ▶); program(s) used to refine structure: *SHELXL97* (Sheldrick, 2008 ▶); molecular graphics: *ORTEP-3* (Farrugia, 1997 ▶) and *DIAMOND* (Brandenburg, 2006 ▶); software used to prepare material for publication: *MarvinSketch* (Chemaxon, 2010 ▶) and *publCIF* (Westrip, 2010 ▶).

## Supplementary Material

Crystal structure: contains datablock(s) I, global. DOI: 10.1107/S1600536811020356/hg5045sup1.cif

Structure factors: contains datablock(s) I. DOI: 10.1107/S1600536811020356/hg5045Isup2.hkl

Supplementary material file. DOI: 10.1107/S1600536811020356/hg5045Isup3.cml

Additional supplementary materials:  crystallographic information; 3D view; checkCIF report

## Figures and Tables

**Table 1:** Hydrogen-bond geometry (Å, °)

*D*—H⋯*A*	*D*—H	H⋯*A*	*D*⋯*A*	*D*—H⋯*A*
C2—H2⋯O3^i^	1.00	2.60	3.5142 (15)	153
C8—H8⋯O2^ii^	0.95	2.60	3.2689 (16)	128
C11—H11b⋯O2^iii^	0.99	2.54	3.3675 (16)	141
C12—H12a⋯O2^iii^	0.98	2.57	3.5448 (16)	173

Symmetry codes: (i) 
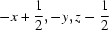
; (ii) 
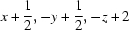
; (iii) 
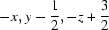
.

**Fig. 1.**
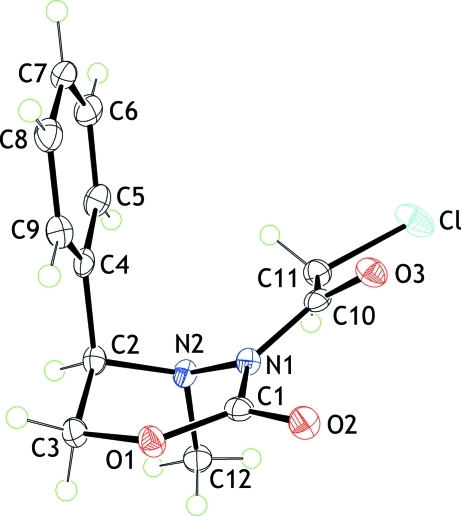
The molecular structure of (I) showing atom labelling scheme and displacement ellipsoids at the 50% probability level (arbitrary spheres for the H atoms).

**Fig. 2.**
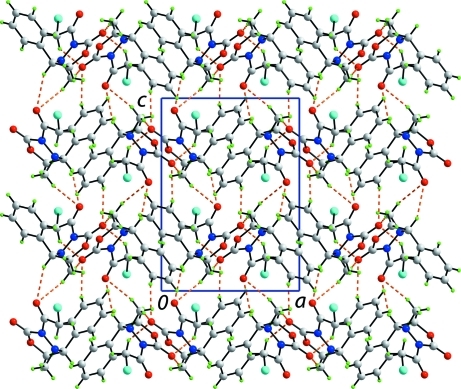
A view of the unit-cell contents in (I) viewed in projection down the b axis showing the C—H···O interactions as orange dashed lines.

## supplementary crystallographic information

### Comment

About forty years ago Trepanier and collaborators reported the first synthesis of some 3,4,5,6-tetrahydro-2*H*-1,3,4-oxadiazin-2-ones as candidates for central nervous system stimulant activity (Trepanier *et al.*, 1968). After three decades from the discovery of 1,3,4-oxadiazin-2-ones, Husson, Micouin and co-workers (Roussi *et al.*, 1998) successfully employed this class of compound as chiral auxiliaries in diastereoselective alkylations and in dipolar cycloadditions (Roussi *et al.*, 1999, 2000, Bonin *et al.*, 2006). In a different approach, Hitchcock and collaborators have been successfully applying 1,3,4-oxadiazinan-2-one derivatives as chiral auxiliaries in asymmetric aldol addition reactions (Casper *et al.*, 2002*b*). Besides their applications in asymmetric synthesis, this class of compounds show a very interesting conformational behaviour (Casper *et al.*, 2002*a*). To explore the adopted conformation of the title compound, (I), in the solid-state, an X-ray study was performed.

The crystal structure analysis of (I) confirms the *R* configuration at atom C2, Fig. 1, in accord with expectation from the synthesis. The 1,3,4-oxadiazinan-2-one ring is in a distorted half-chair conformation, as shown by the ring-puckering parameters: q_2_ = 0.364 (1) Å, q_3_ = -0.333 (1) Å, Q = 493 (1) Å and φ_2_ = 32.0 (2) ° (Cremer and Pople, 1975). The deviations of the O1, C1, N1, N2, C2 and C3 atoms from their least-squares plane are 0.0424 (10) -0.0497 (13), -0.1285 (11), 0.3139 (11), -0.3212 (13) and 0.1430 (13) Å, respectively. The observed conformation contrasts the twisted chair conformation found in the only other single-ring 1,3,4-oxadiazinan-2-one structure known (Zukerman-Schpector *et al.*, 2009). The chloridoacetyl and phenyl groups lie to the same side of the molecule and opposite to that of the N-bound methyl group. The dihedral angles formed between the 1,3,4-oxadiazinan-2-one ring and the phenyl and chloridoacetyl (Cl,O3,C10,C11) groups are 87.69 (6) and 14.41 (3) °, respectively, consistent with axial and equatorial substitution. Molecules are consolidated in the crystal packing by C—H···O interactions operating in three-dimensions, Table 1 and Fig. 2.

### Experimental

The starting (*R*)-4-methyl-5-phenyl-1,3,4-oxadiazinan-2-one was synthesized by using the same procedure as previously reported (Rodrigues *et al.* 2005). The chlorooacetyl-1,3,4-oxadiazinan-2-one (I) species was prepared by the acylation reaction of 1,3,4-oxadiazinan-2-one. A solution of BuLi (2.00 *M* in hexane, 1.45 ml, 2.86 mmol) was added drop wise to a solution of 1,3,4-oxadiazinan-2-one (500 mg, 2.60 mmol) in dry THF (10 ml) at 195 K and the reaction was stirred for an additional 15 min. A chloroacetyl chloride (230 µ*L*, 2.86 mmol) solution in THF (1 ml) was added slowly to the reaction mixture. After 15 min, the light-yellow solution was warmed to RT for a further 30 min. The reaction was quenched with saturated aqueous ammonium chloride solution (5 ml). The mixture was concentrated under reduced pressure, taken up in water (5 ml) and extracted with DCM (3 times; 15 ml) then dried (MgSO_4_). Evaporation of the solvent *in vacuo* gave the crude product which was purified by flash column chromatography on silica gel with 40% EtOAc in hexanes to give the pure product as a colourless solid (572 mg, 82%). Colourless crystals of (I) were obtained by vapour diffusion from hexane/acetone at 298 K. mp = 408 – 410 K; [α]_D_^25^ +52,5° (*c* 1,02, CHCl_3_); ^1^H NMR (500 MHz, CDCl_3_/TMS), δ (p.p.m.): 7.40–7.35 (m, 5H), 4.88 (dd, ^2^*J* = 11.5 Hz, ^3^*J* = 5.3 Hz, 1H), 4.77 (dd, ^2^*J* = 11.5 Hz, ^3^*J* = 7.7 Hz, 1H), 4.43 (*AB* spin system, Dn = 36.0 Hz, ^2^*J* = 15.3 Hz, 2H), 4.40 (dd, ^3^*J* = 7.7 Hz, ^3^*J* = 5.3 Hz, 1H), 2.81 (s, 3H). ^1^H NMR (125 MHz, CDCl_3_/TMS), δ (p.p.m.): 166.07, 149.54, 134.69, 129.16, 128.90, 127.02, 67.65, 61.54, 44.75, 42.12. Anal. calcd for C_12_H_13_ClN_2_O_3_: C, 53.64%; H, 4.88%; N, 10.43%. Found: C, 53.46%; H, 4.92%; N, 10.49%.

### Refinement

Carbon-bound H-atoms were placed in calculated positions (C—H 0.95 to 1.00 Å) and were included in the refinement in the riding model approximation, and with *U_iso_*(H) = 1.2*U_eq_*(C) and 1.*5U*_eq_(methyl-C).

### Crystal data

**Table:**

C_12_H_13_ClN_2_O_3_	*F*(000) = 560
*M**_r_* = 268.69	*D*_x_ = 1.476 Mg m^−^^3^
Orthorhombic, *P*2_1_2_1_2_1_	Mo *K*α radiation, λ = 0.71073 Å
Hall symbol: P 2ac 2ab	Cell parameters from 2003 reflections
*a* = 9.4862 (2) Å	θ = 2.8–16.3°
*b* = 9.6237 (2) Å	µ = 0.32 mm^−^^1^
*c* = 13.2433 (3) Å	*T* = 100 K
*V* = 1209.01 (5) Å^3^	Block, colourless
*Z* = 4	0.35 × 0.30 × 0.25 mm

### Data collection

**Table:**

Bruker APEXII CCD diffractometer	2334 reflections with *I* > 2σ(*I*)
Radiation source: fine-focus sealed tube	*R*_int_ = 0.026
graphite	θ_max_ = 26.0°, θ_min_ = 2.6°
ω scans	*h* = −11→11
29813 measured reflections	*k* = −11→11
2378 independent reflections	*l* = −16→16

### Refinement

**Table:**

Refinement on *F*^2^	Secondary atom site location: difference Fourier map
Least-squares matrix: full	Hydrogen site location: inferred from neighbouring sites
*R*[*F*^2^ > 2σ(*F*^2^)] = 0.020	H-atom parameters constrained
*wR*(*F*^2^) = 0.054	*w* = 1/[σ^2^(*F*_o_^2^) + (0.028*P*)^2^ + 0.320*P*] where *P* = (*F*_o_^2^ + 2*F*_c_^2^)/3
*S* = 1.07	(Δ/σ)_max_ = 0.001
2378 reflections	Δρ_max_ = 0.19 e Å^−^^3^
164 parameters	Δρ_min_ = −0.20 e Å^−^^3^
0 restraints	Absolute structure: Flack (1983), 993 Friedel pairs
Primary atom site location: structure-invariant direct methods	Flack parameter: 0.01 (5)

### Special details

**Table:**

Geometry. All s.u.'s (except the s.u. in the dihedral angle between two l.s. planes) are estimated using the full covariance matrix. The cell s.u.'s are taken into account individually in the estimation of s.u.'s in distances, angles and torsion angles; correlations between s.u.'s in cell parameters are only used when they are defined by crystal symmetry. An approximate (isotropic) treatment of cell s.u.'s is used for estimating s.u.'s involving l.s. planes.
Refinement. Refinement of *F*^2^ against ALL reflections. The weighted *R*-factor *wR* and goodness of fit *S* are based on *F*^2^, conventional *R*-factors *R* are based on *F*, with *F* set to zero for negative *F*^2^. The threshold expression of *F*^2^ > 2σ(*F*^2^) is used only for calculating *R*-factors(gt) *etc*. and is not relevant to the choice of reflections for refinement. *R*-factors based on *F*^2^ are statistically about twice as large as those based on *F*, and *R*- factors based on ALL data will be even larger.

### Fractional atomic coordinates and isotropic or equivalent isotropic displacement parameters (Å^2^)

**Table:**

	*x*	*y*	*z*	*U*_iso_*/*U*_eq_	
Cl	0.24739 (4)	−0.34782 (3)	0.91280 (2)	0.02998 (10)	
O1	0.05085 (9)	0.22385 (9)	0.73203 (7)	0.0182 (2)	
O2	−0.07381 (9)	0.09934 (10)	0.83862 (7)	0.0196 (2)	
O3	0.09385 (10)	−0.08909 (10)	0.93619 (7)	0.0200 (2)	
N1	0.13328 (11)	0.00514 (11)	0.77991 (8)	0.0146 (2)	
N2	0.22794 (11)	0.00168 (11)	0.69603 (7)	0.0156 (2)	
C1	0.02961 (13)	0.10834 (13)	0.78683 (9)	0.0153 (2)	
C2	0.28807 (12)	0.14314 (13)	0.68462 (9)	0.0165 (2)	
H2	0.3517	0.1421	0.6244	0.020*	
C3	0.16885 (13)	0.24374 (14)	0.66273 (9)	0.0184 (3)	
H3A	0.2041	0.3402	0.6689	0.022*	
H3B	0.1359	0.2301	0.5925	0.022*	
C4	0.37768 (13)	0.17604 (13)	0.77675 (9)	0.0163 (2)	
C5	0.49224 (13)	0.08997 (14)	0.79745 (10)	0.0200 (3)	
H5	0.5116	0.0132	0.7545	0.024*	
C6	0.57847 (14)	0.11524 (15)	0.88025 (11)	0.0228 (3)	
H6	0.6556	0.0553	0.8942	0.027*	
C7	0.55191 (14)	0.22803 (15)	0.94258 (10)	0.0221 (3)	
H7	0.6113	0.2458	0.9988	0.027*	
C8	0.43859 (15)	0.31474 (13)	0.92274 (10)	0.0212 (3)	
H8	0.4203	0.3921	0.9654	0.025*	
C9	0.35165 (14)	0.28841 (13)	0.84028 (10)	0.0185 (3)	
H9	0.2737	0.3477	0.8272	0.022*	
C10	0.15311 (13)	−0.09421 (13)	0.85593 (9)	0.0157 (2)	
C11	0.25837 (14)	−0.20626 (13)	0.82638 (9)	0.0194 (3)	
H11A	0.3550	−0.1674	0.8271	0.023*	
H11B	0.2378	−0.2394	0.7571	0.023*	
C12	0.15380 (14)	−0.04923 (14)	0.60530 (9)	0.0195 (3)	
H12A	0.1286	−0.1472	0.6146	0.029*	
H12B	0.2155	−0.0399	0.5464	0.029*	
H12C	0.0680	0.0056	0.5947	0.029*	

### Atomic displacement parameters (Å^2^)

**Table:**

	*U*^11^	*U*^22^	*U*^33^	*U*^12^	*U*^13^	*U*^23^
Cl	0.0483 (2)	0.01763 (15)	0.02398 (16)	0.00875 (16)	−0.00658 (16)	0.00243 (12)
O1	0.0178 (4)	0.0164 (4)	0.0205 (4)	0.0022 (4)	0.0018 (4)	0.0041 (4)
O2	0.0179 (4)	0.0206 (5)	0.0202 (4)	0.0031 (4)	0.0031 (4)	0.0011 (4)
O3	0.0249 (5)	0.0204 (4)	0.0148 (4)	0.0010 (4)	−0.0004 (4)	−0.0004 (4)
N1	0.0149 (5)	0.0139 (5)	0.0150 (5)	−0.0001 (4)	0.0019 (4)	−0.0008 (4)
N2	0.0170 (5)	0.0159 (5)	0.0138 (5)	−0.0026 (4)	0.0035 (4)	−0.0031 (4)
C1	0.0170 (6)	0.0150 (6)	0.0137 (5)	0.0002 (5)	−0.0030 (5)	−0.0017 (5)
C2	0.0177 (6)	0.0157 (6)	0.0162 (5)	−0.0024 (5)	0.0035 (4)	0.0002 (5)
C3	0.0196 (6)	0.0191 (6)	0.0164 (6)	−0.0026 (5)	0.0014 (5)	0.0025 (5)
C4	0.0157 (6)	0.0163 (6)	0.0170 (5)	−0.0050 (5)	0.0035 (5)	0.0016 (5)
C5	0.0160 (6)	0.0195 (6)	0.0245 (7)	−0.0014 (5)	0.0050 (5)	−0.0024 (5)
C6	0.0142 (6)	0.0263 (7)	0.0280 (7)	−0.0014 (5)	0.0016 (5)	0.0039 (6)
C7	0.0200 (6)	0.0271 (7)	0.0193 (6)	−0.0099 (5)	−0.0015 (5)	0.0034 (5)
C8	0.0270 (7)	0.0181 (6)	0.0186 (6)	−0.0052 (5)	0.0019 (5)	−0.0011 (5)
C9	0.0210 (6)	0.0153 (6)	0.0192 (6)	−0.0001 (5)	0.0029 (5)	0.0012 (5)
C10	0.0169 (6)	0.0141 (6)	0.0160 (6)	−0.0031 (5)	−0.0046 (5)	−0.0024 (5)
C11	0.0210 (6)	0.0156 (6)	0.0218 (6)	0.0012 (5)	−0.0027 (5)	0.0017 (5)
C12	0.0233 (7)	0.0199 (6)	0.0154 (6)	−0.0024 (5)	−0.0006 (5)	−0.0034 (5)

### Geometric parameters (Å, °)

**Table:**

Cl—C11	1.7823 (13)	C4—C5	1.3937 (18)
O1—C1	1.3428 (15)	C5—C6	1.3895 (19)
O1—C3	1.4601 (15)	C5—H5	0.9500
O2—C1	1.2001 (15)	C6—C7	1.387 (2)
O3—C10	1.2034 (16)	C6—H6	0.9500
N1—C1	1.4006 (16)	C7—C8	1.386 (2)
N1—C10	1.4012 (16)	C7—H7	0.9500
N1—N2	1.4287 (14)	C8—C9	1.3917 (19)
N2—C12	1.4760 (15)	C8—H8	0.9500
N2—C2	1.4838 (16)	C9—H9	0.9500
C2—C3	1.5167 (18)	C10—C11	1.5209 (17)
C2—C4	1.5203 (17)	C11—H11A	0.9900
C2—H2	1.0000	C11—H11B	0.9900
C3—H3A	0.9900	C12—H12A	0.9800
C3—H3B	0.9900	C12—H12B	0.9800
C4—C9	1.3923 (18)	C12—H12C	0.9800
			
C1—O1—C3	124.28 (10)	C7—C6—C5	119.99 (12)
C1—N1—C10	122.08 (11)	C7—C6—H6	120.0
C1—N1—N2	120.58 (10)	C5—C6—H6	120.0
C10—N1—N2	117.28 (10)	C8—C7—C6	119.96 (12)
N1—N2—C12	109.95 (9)	C8—C7—H7	120.0
N1—N2—C2	107.42 (9)	C6—C7—H7	120.0
C12—N2—C2	113.88 (10)	C7—C8—C9	119.92 (13)
O2—C1—O1	119.42 (11)	C7—C8—H8	120.0
O2—C1—N1	124.07 (12)	C9—C8—H8	120.0
O1—C1—N1	116.50 (10)	C8—C9—C4	120.70 (12)
N2—C2—C3	108.57 (10)	C8—C9—H9	119.7
N2—C2—C4	108.92 (10)	C4—C9—H9	119.7
C3—C2—C4	115.94 (11)	O3—C10—N1	122.96 (12)
N2—C2—H2	107.7	O3—C10—C11	124.27 (12)
C3—C2—H2	107.7	N1—C10—C11	112.77 (11)
C4—C2—H2	107.7	C10—C11—Cl	109.77 (9)
O1—C3—C2	111.58 (10)	C10—C11—H11A	109.7
O1—C3—H3A	109.3	Cl—C11—H11A	109.7
C2—C3—H3A	109.3	C10—C11—H11B	109.7
O1—C3—H3B	109.3	Cl—C11—H11B	109.7
C2—C3—H3B	109.3	H11A—C11—H11B	108.2
H3A—C3—H3B	108.0	N2—C12—H12A	109.5
C9—C4—C5	118.75 (12)	N2—C12—H12B	109.5
C9—C4—C2	123.20 (12)	H12A—C12—H12B	109.5
C5—C4—C2	118.04 (12)	N2—C12—H12C	109.5
C6—C5—C4	120.68 (12)	H12A—C12—H12C	109.5
C6—C5—H5	119.7	H12B—C12—H12C	109.5
C4—C5—H5	119.7		
			
C1—N1—N2—C12	−74.26 (13)	C3—C2—C4—C9	1.96 (17)
C10—N1—N2—C12	108.57 (12)	N2—C2—C4—C5	59.96 (14)
C1—N1—N2—C2	50.16 (13)	C3—C2—C4—C5	−177.28 (11)
C10—N1—N2—C2	−127.01 (11)	C9—C4—C5—C6	0.36 (19)
C3—O1—C1—O2	−176.89 (11)	C2—C4—C5—C6	179.64 (11)
C3—O1—C1—N1	3.76 (17)	C4—C5—C6—C7	−0.77 (19)
C10—N1—C1—O2	−22.68 (18)	C5—C6—C7—C8	0.57 (19)
N2—N1—C1—O2	160.29 (11)	C6—C7—C8—C9	0.02 (19)
C10—N1—C1—O1	156.64 (11)	C7—C8—C9—C4	−0.42 (19)
N2—N1—C1—O1	−20.39 (16)	C5—C4—C9—C8	0.23 (19)
N1—N2—C2—C3	−61.59 (12)	C2—C4—C9—C8	−179.00 (11)
C12—N2—C2—C3	60.42 (12)	C1—N1—C10—O3	−8.18 (18)
N1—N2—C2—C4	65.49 (12)	N2—N1—C10—O3	168.94 (11)
C12—N2—C2—C4	−172.50 (10)	C1—N1—C10—C11	172.73 (11)
C1—O1—C3—C2	−18.86 (16)	N2—N1—C10—C11	−10.15 (15)
N2—C2—C3—O1	47.44 (13)	O3—C10—C11—Cl	14.29 (16)
C4—C2—C3—O1	−75.51 (14)	N1—C10—C11—Cl	−166.64 (8)
N2—C2—C4—C9	−120.81 (13)		

### Hydrogen-bond geometry (Å, °)

**Table:**

*D*—H···*A*	*D*—H	H···*A*	*D*···*A*	*D*—H···*A*
C2—H2···O3^i^	1.00	2.60	3.5142 (15)	153
C8—H8···O2^ii^	0.95	2.60	3.2689 (16)	128
C11—H11b···O2^iii^	0.99	2.54	3.3675 (16)	141
C12—H12a···O2^iii^	0.98	2.57	3.5448 (16)	173

Symmetry codes: (i) −*x*+1/2, −*y*, *z*−1/2; (ii) *x*+1/2, −*y*+1/2, −*z*+2; (iii) −*x*, *y*−1/2, −*z*+3/2.
